# Knowledge, attitudes, and practices of breastfeeding among women visiting primary healthcare clinics on the island of Abu Dhabi, United Arab Emirates

**DOI:** 10.1186/s13006-018-0165-x

**Published:** 2018-07-03

**Authors:** Mai Isam Al Ketbi, Sultan Al Noman, Abdelqadir Al Ali, Ebtihal Darwish, Maha Al Fahim, Jaishen Rajah

**Affiliations:** 1 Sheikh Khalifa Medical City (SKMC), P.O. Box: 51900, Abu Dhabi, United Arab Emirates; 2 Ethraa Consultation and Training, P.O. Box: 32311, Dubai, United Arab Emirates

**Keywords:** Abu Dhabi Island, U.A.E., Breastfeeding knowledge, Breastfeeding attitude, Breastfeeding practice, Breastfeeding duration, Reasons for stopping breastfeeding, Breastfeeding, Exclusive breastfeeding, Primary healthcare clinics

## Abstract

**Background:**

The World Health Organization recommends continued breastfeeding up to 2 years of age or beyond. This study assessed breastfeeding knowledge, attitudes, and practices among women residing on the island of Abu Dhabi and identified associated factors.

**Methods:**

We conducted a cross-sectional study using a self-administered questionnaire among mothers visiting primary healthcare clinics in Abu Dhabi between November 2014 and 2015. Participants were women aged at least 18 years who had at least one child aged 2 years or younger at the time of the study. Breastfeeding knowledge, attitudes, and practices were assessed on the basis of experience with last child. Selected questions were used to develop a scaled scoring system to categorize these aspects as good, fair, or poor. Exclusive breastfeeding is defined as the act of feeding infants only breast milk since birth, without providing water, formula, or other liquid supplements.

**Results:**

The participants were 344 women. Exclusive breastfeeding for 6 months was reported by only 46 (16.9%, 95% CI 0.10, 0.17, *n* = 272). 79 (28.7%, *n* = 275) of the participants were breastfeeding and planning to continue after the child was ≥24 months. Multivariate logistic regression analysis revealed that the following factors were associated with exclusive breastfeeding: mothers with female children (adjusted OR [AOR] 2.42; 95% CI 1.18, 4.97) and better breastfeeding knowledge scores (AOR 1.25; 95% CI 1.04, 1.50). The following factors were associated with less likelihood of exclusively breastfeeding: working mothers (AOR 0.29; 95% CI 0.12, 0.72), living with relatives (AOR 0.21; 95% CI 0.05, 0.81), no past exclusive breastfeeding experience (AOR 0.23; 95% CI 0.09, 0.58) and being offered readymade liquid formula in hospital (AOR 0.33; 95% CI 0.15, 0.72). The most common reason for stopping breastfeeding was insufficient breast milk production (68/89, 76%), and the most common work related reason was inadequate maternity leave (24/89, 15%).

**Conclusion:**

Although breastfeeding knowledge was generally good, breastfeeding practice was still suboptimal. Modifiable factors found to predict exclusive breastfeeding included breastfeeding knowledge and mothers’ employment status.

**Electronic supplementary material:**

The online version of this article (10.1186/s13006-018-0165-x) contains supplementary material, which is available to authorized users.

## Background

The importance of breastfeeding for both infants and mothers are globally recognized [1]. Exclusive breastfeeding is defined as the act of feeding the infant only breast milk, with no supplemental liquids or solids except for liquid medicine or vitamin/mineral supplements [2]. During the first 6 months of life, breast milk alone is the ideal nourishment for infants, providing all the necessary nutrients, including vitamins and minerals [3].

The American Academy of Pediatrics (AAP) recommends exclusive breastfeeding for 6 months and continued breastfeeding for at least 12 months; thereafter, it can be continued for as long as the mother and the baby desire [1]. The World Health Organization (WHO) recommends continued breastfeeding up to 2 years of age or beyond [4] and it has been estimated that optimal breastfeeding of children younger than 2 years, could annually save the lives of over 800,000 children under 5 years of age [4].

Using data from 66 countries, a review of the differences in the prevalence of exclusive breastfeeding among infants younger than 6 months between 1995 and 2010 revealed that the prevalence of exclusive breastfeeding among infants of this age group in developing countries increased from 33% in 1995 to merely 39% in 2010 [5]. Despite considerable improvements in some regions, the prevalence of exclusive breastfeeding remains far too low in many areas of the developing world [5].

The knowledge, attitudes, and practices among women regarding breastfeeding vary in different countries. In the United Arab Emirates (U.A.E.), a recent study involving 593 Emirati mothers showed that the feeding practices of infants and young children were suboptimal [6]. Although almost all the mothers in the study had initiated breastfeeding (98%), only 25% of the infants had been exclusively breastfed since birth at 6 months of age [6]. In another prospective study conducted in Sharjah, U.A.E. [7], a sample of 221 women who gave birth at Al Qassimi Hospital were surveyed regarding breastfeeding patterns at 1 day, 1 month, and 6 months postpartum; the corresponding exclusive breastfeeding rates since birth were 76.5, 48.4, and 13.3%, respectively [7]. Unfortunately, local data on the knowledge, attitudes, and practices among women regarding breastfeeding are limited.

In Saudi Arabia, a recent cross-sectional study on school teachers in the Abha female educational district showed that out of 384 women, 31% started breastfeeding their children within 1 hour of delivery, while only 8.3% reported exclusive breastfeeding for 6 months [8]. Regarding their knowledge, although 89.3% of the participants reported that colostrum is good for the baby, there was a low rate of knowledge regarding the appropriate duration of exclusive breastfeeding: only 28% of the participants chose 6 months as the answer, and this might explain the low rate of exclusive breastfeeding at 6 months [8]. Regarding their attitudes, the most important reason given by the participants for initiating breastfeeding was their Islamic religious background (56.6%), which was practiced by all of them [8].

In January 2014, the Federal National Council’s (FNC) Health, Labour and Social Affairs committee proposed adding a breastfeeding clause to the U.A.E.’s Child Rights Law, which would make breastfeeding a mandatory requirement for mothers of all children up to the age of 2 years as breastfeeding is crucial for a child’s development [9]. This proposed breastfeeding clause has not yet been officially passed. Fortunately, a new law extending fully paid maternity leave from 2 months to 3 months was recently established and passed in the U.A.E., which is a step in the right direction [10]. However, there is more to be done since this law is applied to government entities only and hence not all private companies have chosen to implement this new policy [10, 11].

Despite WHO recommendations, it was observed that not many women in the U.A.E were breastfeeding exclusively for 6 months since birth of the infant [6, 7]. Since there is limited local data on the knowledge and attitudes of women toward breastfeeding in the U.A.E., research in this field is vital to explore the knowledge, attitudes, and practices regarding breastfeeding among women and to explore any possible barriers to exclusive breastfeeding so as to develop strategies for raising awareness, overcome any barriers, and improve breastfeeding rates. Therefore, the aim of our study is to assess breastfeeding knowledge, attitudes, and practices among women residing in Abu Dhabi and to identify factors that influence these aspects of breastfeeding.

## Methods

### Study setting and population

This cross-sectional study was conducted among mothers visiting four primary healthcare clinics, Bateen, Zafaranah, Khaleej, and Rowda, in Abu Dhabi between November 2014 and November 2015 using a self-administered questionnaire.

The target study population was composed of women aged 18 years or older, regardless of nationality, who had at least one child aged 2 years or younger at the time of the study. We excluded women who were non-English or non-Arabic speakers. The estimated target population size was 4470, based on the clinics’ attendance records during January. Based on the null hypothesis that 13% of population is exclusively breastfeeding for 6 months, the sample size was calculated using a 95% confidence interval (Newcombe formula) and was found to be 354 mothers (http://www.surveysystem.com/sscalc.htm) [12, 13].

The self-administered questionnaires were then proportionally distributed to the four primary health care clinics based on the number of patients attending each clinic from the total sample size. At each clinic, questionnaires were distributed to women who met the inclusion and exclusion criteria in a convenience non-random collection sampling until the required numbers were attained. The aim was to assess the participants’ breastfeeding knowledge, attitudes, and practices on the basis of their experience with their last child. In this study, exclusive breastfeeding was defined as feeding of infants with only breast milk since birth, with no water, formula or liquid supplements.

### Study instrument

Given the absence of a validated standardized questionnaire, the questionnaire used in the present study was adapted from a similar study that was conducted in Saudi Arabia [8] and modified to meet our objectives.

The resulting self-administered questionnaire contained a total of 50 questions and was divided into four parts that addressed the participants’ sociodemographic characteristics (13 questions) and breastfeeding knowledge (13 questions), attitudes (7 questions), and practices (13 questions). The majority of the questions were based on the participants’ experience with their last child unless otherwise specified. Selected questions under breastfeeding knowledge (12 questions), attitudes (6 questions), and practices (10 questions) were used to develop a scaled scoring system to categorize these aspects as good, fair, or poor. Each correct or favourable answer chosen by participants was given one or two points; then the points were summed for each participant. No points were deducted for wrong or unfavourable answers. The participants’ scoring system was as follows:

- Knowledge: Good (8–12 points), Fair (4–7 points), Poor (0–3 points)
- Attitude: Good (5–6 points), Fair (3–4 points), Poor (0–2 points)
- Practice: Good (4–10 points), Fair (2–3 points), Poor (0–1 point)

Questions intended to assess breastfeeding knowledge included the average number of feedings a child should receive per day, duration of breastfeeding from each breast, importance of colostrum, benefits of breastfeeding to the child and the mother, age up to which the child should receive breast milk, age at which the mother should start supplementary food, whether breast milk alone is better than formula milk to fulfill the child’s necessary dietary requirements, sufficiency of breast milk alone during the first 6 months of life, relationship between breastfeeding and the mother’s weight, whether breast milk loses its benefits when it is pumped, and storage duration for pumped breast milk at room temperature and in a refrigerator.

Questions intended to assess breastfeeding attitudes included the reasons for adopting breastfeeding, participant self-image (weight and hair loss), whether breast milk loses its benefits when it is pumped, whether breastfeeding should be stopped because of medications, intention to breastfeed future children, and intention to participate in classes related to breastfeeding for future pregnancies.

Questions intended to assess breastfeeding practices included the child’s age at which breastfeeding was stopped, reasons for stopping breastfeeding before the child reached 2 years of age, time of breastfeeding initiation after delivery, duration of exclusive breastfeeding since birth, age of starting formula and other supplements, whether the child was given ready-made liquid formula in the hospital, and whether the mother attended classes related to breastfeeding during pregnancy. Questions used to measure exclusive breastfeeding since birth among participants included:

- For how long did you exclusively breastfeed (giving your child only breast milk, no formula milk or food) your last child since birth?
- If you are still exclusively breastfeeding (giving your child only breast milk, no formula milk or food) your last child, until what age are you planning to continue?

Informed consent forms were attached with each questionnaire for the participants to read and sign if they were willing to participate in the study. Questionnaires and informed consent forms were drafted in English and Arabic. A pilot study was conducted after ethical approval was granted in order to assess the questionnaire’s comprehensibility, and modifications were accordingly made. The pilot sample met this study’s inclusion and exclusion criteria and the pilot sample size was 15.

The charge nurses positioned at the four primary healthcare clinics were briefed regarding the questions included in the questionnaire and given training regarding the participants’ anonymity and informed consent.

### Data collection

Questionnaires were printed and then proportionally distributed to the four primary healthcare clinics in Abu Dhabi. The charge nurse at each clinic was requested to distribute the questionnaires randomly to patients who matched our inclusion criteria. After the completion of the questionnaires by the participants, the charge nurses collected and sealed the questionnaires in envelopes to ensure the participants’ confidentiality. After the end of the study period, the charge nurses were requested to return the completed questionnaires to the authors. On the basis of the participants’ responses, their knowledge, attitudes, and practices of breastfeeding were assessed.

### Data analysis

After the collection of the questionnaires, the obtained data were organized using the MS Excel software program, coded, and analyzed using the Statistical Package for Social Sciences (SPSS) version 16. Means and standard deviation (SD) were used for numerical data, whereas percentages were used for categorical data.

First, chi squared (χ^2^) test was conducted to assess the effect of certain factors on breastfeeding knowledge, attitudes, and practices (specifically exclusive breastfeeding). Factors that were analyzed in this study included: mother’s age, mother’s educational background, mother’s employment status, employment sector, entitlement to breastfeeding hours by employer, living with husband and children only or along with relatives, number of housemaids or nannies, monthly family income, number of children, gender of last child, last child’s gestational age at delivery, mode of delivery of last child, healthcare provider explained the importance of breastfeeding during antenatal visits for last pregnancy, healthcare provider explained the importance of breastfeeding after delivery of last child, healthcare provider explained the appropriate practices of breastfeeding for last child, past breastfeeding experience and past exclusive breastfeeding experience.

Second, association between the dependent variable (exclusive breastfeeding for 6 months = 1 or not = 0) and independent variables was estimated in multivariate logistic regression analysis using MedCalc Statistical Software version 18 (MedCalc Software bvba, Ostend, Belgium; http://www.medcalc.org; 2018). The number of variables selected in our study is based on the recommendations of approximately ten variables per event to prevent bias in both directions [14, 15]. Adjusted odds ratio (AOR) with 95% confidence interval (CI) were computed and a *p* - value less than 0.05 was selected as the cutoff for statistical significance.

## Results

A total of 354 questionnaires were distributed, but only 344 participants returned completed questionnaire copies, yielding a response rate of 97%.

### Description and characteristics of participants

#### Mother-related

Most of the participants in the study sample were aged 25–29 years (37.5%), had 2–4 children (60.6%) had university or higher educational degrees (80%), and the mean (SD) duration of past breastfeeding experience was 16.2 months (± 7.3) (Tables 1 and 2).

**Table 1:** Selected characteristics of the participants in the study sample (*n* = 344)

Variable	Number	(%)
Age (in years)		
18–24	39	(11.4)
25–29	128	(37.5)
30–34	107	(31.4)
35–39	57	(16.7)
40–44	10	(3)
≥ 45	0	(0)
Marital status		
Married	338	(99.1)
Divorced	2	(0.6)
Widowed	1	(0.3)
Educational background		
Primary school or lower	3	(0.9)
Secondary school	65	(19.1)
University or higher	272	(80)
Employed		
No	237	(69.3)
Yes	105	(30.7)
Self-employed	0	(0)
Employment sector		
Private	68	(66)
Public	35	(34)
Entitled to breastfeeding hours by employer		
Yes	83	(82.2)
No	18	(17.8)
Living with husband and children only		
Yes	293	(86.4)
No (Living with relatives)	43	(12.7)
No (Separated/Divorced/Widowed)	3	(0.9)
Number of housemaids or nannies		
0	240	(73.2)
1	70	(21.3)
> 1	18	(5.5)
Monthly family income (in AED)		
< 15,000	137	(42)
15,000–30,000	153	(46.9)
> 30,000	36	(11.1)
Number of children		
1	122	(35.9)
2–4	206	(60.6)
≥ 5	12	(3.5)
Gender of last child		
Male	169	(51.4)
Female	160	(48.6)
Last child’s gestational age at delivery		
< 37 weeks	69	(20.5)
≥ 37 weeks	267	(79.5)
Mode of delivery of last child		
Vaginal delivery	196	(57.6)
Caesarian section	144	(42.4)
Healthcare provider explained the importance of breastfeeding during antenatal visits for last pregnancy		
Yes	275	(81.6)
No	62	(18.4)
Healthcare provider explained the importance of breastfeeding after delivery of last child		
Yes	286	(84.4)
No	53	(15.6)
Healthcare provider explained the appropriate practices of breastfeeding for last child		
Yes	268	(79.8)
No	68	(20.2)

**Table 2:** Participants’ characteristics in the study sample: Mean (± SD) and Median (± IQR) (*n* = 344)

Variable	Mean (± SD^a^)	Median (±IQR^b^)
Number of people living inside house (Including housemaids, drivers, etc.)	4.7 (± 2.9)	4 (± 2)
Age of last child (in months)	8.2 (± 5.7)	6 (± 8)
Employer-entitled breastfeeding hours per day (in hours)	1.1 (± 0.3)	1 (± 1)
Employer-entitled breastfeeding hours duration (in months)	15.3 (± 5.2)	18 (± 6)
Breastfeeding duration of previous children (in months) (*n* = 171)	16.2 (± 7.3)	17 (± 12)
Age of stopping breastfeeding of last child (in months) (*n* = 66)	7.1 (± 5.4)	6 (± 9)

^a^*SD* Standard deviation, ^b^ *IQR* Interquartile range

#### Family and child -related

The majority of the participants had a monthly family income of 15,000–30,000 U.A.E. dirham (AED; 46.9%) (Table 1). The median (IQR) number of people living in the participants’ houses (including housemaids, drivers, etc.) was 4 (± 2) (Table 2)*.* The mean (SD) age of the participants’ last child was 8.2 months (± 5.7), and the mean (SD) breastfeeding duration of that child was 7.1 months (± 5.4) (Table 2).

#### Employer-related

The majority of the participants were entitled to breastfeeding hours by their employer (82.2%) (Table 1). The mean (SD) employer-entitled breastfeeding hours per day was 1.1 h (± 0.3), and the mean (SD) duration of the right to these employer-entitled breastfeeding hours was 15.3 months (±5.2) (Table 2).

### Mothers’ breastfeeding knowledge

A total of 176 (51.2%) mothers were found to have good breastfeeding knowledge, 149 (43.3%) had fair knowledge, and only 19 (5.5%) had poor knowledge (Table 3). Among the mothers, 273 (81.2%) reported that breast milk is sufficient for a child in the first 6 months of life and 290 (86.1%) reported that complementary food should be introduced at 6 months of age. A total of 114 (33.9%) mothers reported that a child should receive breast milk for at least 24 months of age (Table 3). The most common sources of information regarding breastfeeding for participants were family (66.5%), doctors (58.5%), nurses/midwives (50.1%), and the Internet (40.7%) (Table 3).

**Table 3:** Breastfeeding knowledge of participants visiting four primary healthcare clinics in Abu Dhabi (*n* = 344)

Variable	Number	(%)
Correctly answered breastfeeding knowledge questions by participants (total of 12 scored questions):		
Breastfeeding child ≥8 times/day during the first month	179	(53.1)
Breastfeeding duration ≥15 min from each breast during the first month	116	(34.6)
Colostrum is good for child	289	(86)
Breastfeeding is beneficial for both the mother and the child	315	(94)
Children should receive breast milk until ≥24 months of age	114	(33.9)
Complementary food should be introduced at 6 months of age	290	(86.1)
Breast milk is superior to formula milk in fulfilling child’s necessary dietary requirements	323	(96.1)
Breast milk is sufficient for child in the first 6 months of life	273	(81.2)
Breastfeeding decreases the mother’s weight	193	(57.3)
Breast milk does not lose its benefits when it is pumped out or stored	184	(55.6)
Pumped breast milk can be stored at room temperature (60 °F–85 °F/15.5 °C–29.4 °C) for up to 8 hours	160	(47.5)
Pumped breast milk can be stored in the refrigerator (39 °F or colder/3.99 °C or colder) for up to 8 days	99	(29.6)
Participants’ overall knowledge level:		
Good	176	(51.2)
Fair	149	(43.3)
Poor	19	(5.5)
Participants’ sources of information about breastfeeding:		
Doctors	197	(58.5)
Nurses/midwives	169	(50.1)
Friends	111	(32.9)
Family	224	(66.5)
TV programs	80	(23.7)
Campaigns	51	(15.1)
Magazines	49	(14.5)
Breastfeeding classes	52	(15.4)
Internet	137	(40.7)
Others	11	(3.3)

### Factors affecting mothers’ breastfeeding knowledge

Better breastfeeding knowledge was seen among mothers with university or higher educational background (*p* = 0.001), those who were employed (*p* = 0.001), those who lived with their husband and children along with relatives (*p* = 0.000), those who adopted breastfeeding because of their mother or mother-in-law’s encouragement (*p* = 0.005), those with a monthly family income of more than 30,000 AED (*p* = 0.011), and those who only had one child (*p* = 0.016) (Additional file 1).

Statistically significant correlations were also found between better breastfeeding knowledge and having given birth to the last child at 37 weeks or more (*p* = 0.003), having received advice from a healthcare provider about the importance of breastfeeding during antenatal (*p* = 0.049) or postnatal visits (*p* = 0.001), having been counseled about the appropriate breastfeeding practices at any time (*p* = 0.000), having past breastfeeding experience (*p* = 0.002) (Additional file 1).

### Mothers’ breastfeeding attitude

Only 72 (20.9%) mothers were found to have a good breastfeeding attitude, 182 (52.9%) had a fair attitude, and 90 (26.2%) had a poor attitude (Table 4). A total of 307 (92.8%) mothers reported a positive attitude in the form of an intention to breastfeed future children and 219 (66.6%) planned to attend breastfeeding classes for future pregnancies (Table 4). As reasons for adopting breastfeeding, most of the participants reported child health (89.8%), religious background (37%), and cleanliness and easy preparation (31.6%) (Table 4).

**Table 4:** Breastfeeding attitudes of participants visiting four primary healthcare clinics in Abu Dhabi (*n* = 344)

Variable	Number	(%)
Response of participants to breastfeeding attitude questions (Total of six scored questions)		
Breastfeeding can increase the mother’s weight:		
Agree	69	(20.6)
Neutral	86	(25.7)
Disagree^a^	180	(53.7)
One of the causes of hair loss is breastfeeding:		
Agree	114	(34)
Neutral	74	(22.1)
Disagree^a^	147	(43.9)
Pumping breast milk makes it no longer beneficial for the child:		
Agree	39	(11.7)
Neutral	89	(26.7)
Disagree^a^	205	(61.6)
Mothers should stop breastfeeding if they take any type of medication:		
Agree	138	(41.8)
Neutral	105	(31.8)
Disagree^a^	87	(26.4)
Intention to breastfeed future children:		
Agree^a^	307	(92.8)
Neutral	15	(4.5)
Disagree	9	(2.7)
Plan to attend breastfeeding classes in future pregnancy:		
Agree^a^	219	(66.6)
Neutral	82	(24.9)
Disagree	28	(8.5)
Overall participants’ attitude level:		
Good	72	(20.9)
Fair	182	(52.9)
Poor	90	(26.2)
Reasons behind adoption of breastfeeding:		
Religious background	123	(37)
Healthcare providers	92	(27.7)
Child health	298	(89.8)
Media	26	(7.8)
Cleanliness and easy preparation	105	(31.6)
Personal determination or experience	11	(3.3)
Encouragement from mother/mother-in-law	79	(23.8)
Encouragement from husband	81	(24.4)
Other	8	(2.4)
I don’t know	11	(3.3)

^a^Response indicates positive attitude

### Factors affecting mothers’ breastfeeding attitude

Better breastfeeding attitudes were observed among mothers in 35–39 years age group (*p* = 0.003), those employed in the private sector (*p* = 0.017), and those who gave birth to their last child at 37 weeks or later (*p* = 0.005) (Additional file 2).

Statistically significant correlations were also found between better breastfeeding attitudes and living with a husband and children along with other relatives (*p* = 0.014), having one maid or nanny (*p* = 0.016), and having a monthly family income of 15,000–30,000 AED (*p* = 0.005) (Additional file 2).

### Mothers’ breastfeeding practice

Only 94 (27.8%) mothers were found to adopt good breastfeeding practices, 129 (38.2%) adopted fair practices, and 115 (34%) adopted poor practices (Table 5). Although a high percentage of mothers had breastfed their last child for any duration of time (84.4%), had no difficulties in breastfeeding their last child (82.1%), and had past breastfeeding experience and exclusive breastfeeding experience (56.9 and 39.4%, respectively), it was noted that only 79 (28.7%) mothers were currently breastfeeding their last child at the time of the study and were intending to continue at least until the child reached 24 months of age. Only 46 (16.9%) (95% CI 0.10, 0.17) [16] mothers had exclusively breastfed their last child for 6 months and only eight (2.9%) were planning to continue exclusive breastfeeding until the child reached 6 months of age (Table 5).

**Table 5:** Breastfeeding practices of participants visiting four primary healthcare clinics in Abu Dhabi

Variable	Number	(%)	Sample size (n)
Positively answered breastfeeding practice questions by participants on the basis of their experience with their last child (Total of six scored questions):			
Initiation of breastfeeding immediately and within the first hour of life	193	(72.6)	266
Currently breastfeeding the last child and intending to continue until the age of ≥24 months	79	(28.7)	275
Exclusively breastfed last child for 6 months	46	(16.9)	272
Planning to continue exclusively breastfeeding last child until 6 months of age (for children < 6 months of age)	8	(2.9)	273
Child was not given ready-made liquid formula in the hospital	176	(58.3)	302
Attended breastfeeding classes during pregnancy	68	(22.2)	306
Participants’ overall practice level			338
Good	94	(27.8)	
Fair	129	(38.2)	
Poor	115	(34)	
Breastfed last child for any duration of time	266	(84.4)	315
Had no difficulties in breastfeeding last child	220	(82.1)	268
Has past breastfeeding experience from previous children	185	(56.9)	325
Has past exclusive breastfeeding experience from previous children	124	(39.4)	315
Was advised to start formula milk for last baby by the following persons			277
Doctor	133	(48)	
Nurse	3	(1. 1)	
Pharmacist	8	(2.9)	
Family member	30	(10.8)	
Friends	15	(5.4)	
Others	1	(0.4)	
No one	25	(9)	

Feeding practices for the last child were also studied, and it was found that mothers had introduced water, herbal supplements, formula milk, and food at early mean (±SD) ages (4.2 ± 2.1, 3.3 ± 2.9, 3.1 ± 2.9 and 6.1 ± 1.7, respectively). Regarding formula milk use, 176 (58.3%) mothers reported that their last child was not given ready-made liquid formula in the hospital, but most mothers (48%) reported that they were advised by a doctor to start formula milk for their last child (Table 5).

Moreover, non-work related and work related reasons for stopping breastfeeding before the age of 24 months were explored and are summarized in Table 6. Among the 89 women who had stopped breastfeeding, the most common non-work related reasons that women reported were decreased milk production (76.4%), the baby seeming hungry or unsatisfied after feeding (33.3%), and the baby refusing to feed (21.3%). Moreover, 15.7% of mothers reported that they stopped breastfeeding because they had to return to work. The most common work related reason for stopping breastfeeding was insufficient maternity leave, which prevented mothers from developing a breastfeeding schedule or habit (14.5%), followed by insufficient time off during work days for breastfeeding (12%).

**Table 6:** Reasons indicated by mothers who stopped breastfeeding before their children turned two (*n* = 89)

Reasons cited as important	Number	(%)
Lactation factors		
Breast problem (pain, cracked nipple, etc.)	8	(9)
Psychosocial factors		
Breastfeeding was tiring	9	(10.1)
Felt it was time to stop	5	(5.6)
Had too many household duties or other commitments	4	(4.5)
Difficulty finding nursing areas outside the home	4	(4.5)
Breastfeeding was too inconvenient	3	(3.4)
Lack of husband’s support	2	(2.2)
Family recommendation	1	(1.1)
Wanted or needed someone else to feed my baby	0	(0)
Nutritional factors		
Decreased milk production	68	(76.4)
Baby hungry/unsatisfied after feeding	30	(33.3)
Baby not gaining sufficient weight	10	(11.2)
Physician’s recommendation	5	(5.6)
Lifestyle factors		
Decreased food intake by mother	6	(6.7)
The mother’s hair started falling out	2	(2.2)
Breastfeeding was affecting the mother’s shape/body image	1	(1.1)
Medical factors		
Health problems related to the mother (maternal illness/medication use, etc.)	8	(9)
Became pregnant or wanted to become pregnant again	5	(5.6)
Child health problem	0	(0)
Milk-pumping factors		
Pumping milk difficult/time consuming, etc.	4	(4.5)
Infant’s self-weaning factors		
Refused to feed	19	(21.3)
Began to bite	5	(5.6)
Work related factors		
Had to return to work	14	(15.7)
Maternity leave not long enough to develop breastfeeding schedule/habit	24	(14.5)
Insufficient time off during work days for breastfeeding	20	(12)
Home too far from work for breastfeeding	9	(5.4)
Lack of work flexibility for breastfeeding time	9	(5.4)
Lack of nursery at or close to work	8	(4.8)
Could not or did not want to pump or breastfeed at work	8	(4.8)
Problems related to workplace	4	(2.4)
Other reasons	4	(2.4)
Other factors	3	(3.4)

### Factors affecting mothers’ breastfeeding practice and exclusive breastfeeding

Better breastfeeding practice was seen among mothers who delivered their last child by vaginal delivery (*p* = 0.003) and those who had past experience of exclusive breastfeeding (*p* = 0.009) (Additional file 3).

In regards to factors affecting exclusive breastfeeding practices specifically, the following were found to have a statistically significant effect on exclusive breastfeeding: mother’s employment status (*p* = 0.004), living with relatives (*p* = 0.048), gender of last child (*p* = 0.01), gestation of last child (*p* = 0.027), past exclusive breastfeeding experience (*p* = 0.000) and mothers offered readymade liquid formula in hospital (*p* = 0.001). (Table 7).

**Table 7:** Factors that affect exclusive breastfeeding (EBF) practices among participants

Variable	No EBF	EBF	*p*-value
	Number (%)	Number (%)	
Age (in years)			0.362
18–24	13 (72.2)	5 (27.8)	
25–29	58 (69.9)	25 (30.1)	
30–34	50 (67.6)	24 (32.4)	
35–39	32 (78)	9 (22)	
40–44	7 (100)	0 (0)	
≥ 45	0 (0)	0 (0)	
Education background			0.814
Primary school or lower	1 (100)	0 (0)	
Secondary school	29 (70.7)	12 (29.3)	
University or higher	129 (71.7)	51 (28.3)	
Employed			0.004
No	100 (65.8)	52 (34.2)	
Yes	60 (84.5)	11 (15.5)	
Self-employed	0 (0)	0 (0)	
Employment sector			0.052
Private	35 (77.8)	10 (22.2)	
Public	23 (95.8)	1 (4.2)	
Entitled to breastfeeding hours by employer			0.112
Yes	46 (80.7)	11 (19.3)	
No	11 (100)	0 (0)	
Living with husband and children only			0.048
Yes	130 (68.4)	60 (31.6)	
No (Living with relatives)	24 (88.9)	3 (11.1)	
No (Separated/ Divorced/Widowed)	3 (100)	0 (0)	
Number of housemaids or nannies			0.156
0	99 (67.8)	47 (32.2)	
1	41 (82)	9 (18)	
> 1	11 (73.3)	4 (26.7)	
Monthly family income (in AED)			0.209
< 15,000	68 (77.3)	20 (22.7)	
15,000–30,000	71 (65.7)	37 (34.3)	
> 30,000	15 (71.4)	6 (28.6)	
Number of children			0.151
1	56 (78.9)	15 (21.1)	
2–4	95 (66.9)	47 (33.1)	
≥ 5	9 (90)	1 (10)	
Gender of last child			0.010
Male	87 (79.1)	23 (20.9)	
Female	67 (63.2)	39 (36.8)	
Last child gestational age at delivery			0.027
< 37 weeks	36 (85.7)	6 (14.3)	
≥ 37 weeks	123 (68.7)	56 (31.3)	
Mode of delivery of last child			0.550
Vaginal delivery	91 (70.5)	38 (29.5)	
Caesarian section	69 (74.2)	24 (25.8)	
Healthcare provider explained the importance of breastfeeding during antenatal visits for last pregnancy			0.482
Yes	124 (70.9)	51 (29.1)	
No	35 (76.1)	11 (23.9)	
Healthcare provider explained the importance of breastfeeding after delivery of last child			0.631
Yes	133 (72.3)	51(27.7)	
No	26 (68.4)	12 (31.6)	
Healthcare provider explained the appropriate practices of breastfeeding for last child			0.849
Yes	121 (71.2)	49 (28.8)	
No	37 (72.5)	14 (27.5)	
Past breastfeeding experience			0.168
No other children	52 (76.5)	16 (23.5)	
No	12 (85.7)	2 (14.3)	
Yes	91 (66.9)	45 (33.1)	
Past exclusive breastfeeding experience			0.000
No other children	51 (78.5)	14(21.5)	
No	54 (85.7)	9 (14.3)	
Yes	49 (56.3)	38 (43.7)	
Offered readymade formula in hospital			0.001
Yes	72 (84.7)	13 (15.3)	
No	84 (63.2)	49 (36.8)	

### Logistic regression analysis for factors influencing exclusive breastfeeding practices

Factors influencing exclusive breastfeeding practices for 6 months were explored using multivariate logistic regression analysis (Table 8). Only factors that were found to have a statistically significant effect on exclusive breastfeeding in chi squared test were included in the multivariate logistic regression analysis.

**Table 8:** Multivariate logistic regression for the predictors associated with exclusive breastfeeding (*n* = 196)

Variables	Number	Coefficient	Standard Error	Adjusted OR^a^	(95% CI^b^)	*p*-value
Living with husband and children only						
Yes	171			1		
No (Living with relatives)	25	-1.58036	0.69742	0.21	(0.05 , 0.81)	0.0235
Past exclusive breastfeeding experience						
No other children	57	-1.08063	0.44158	0.34	(0.14 , 0.80)	0.0144
No	57	-1.46617	0.47043	0.23	(0.09 , 0.58)	0.0018
Yes	82			1		
Given readymade formula in hospital						
Yes	77	-1.12242	0.40437	0.33	(0.15 , 0.72)	0.0055
No	119			1		
Gender of last child						
Male	101			1		
Female	95	0.88259	0.36800	2.42	(1.18 , 4.97)	0.0165
Breastfeeding knowledge score	196	0.22396	0.092921	1.25	(1.04 , 1.50)	0.0159
Employment status						
No	135			1		
Yes	61	-1.22911	0.45997	0.29	(0.12 , 0.72)	0.0075

^a^*OR* Odds Ratio

^b^*CI* Confidence Interval

The multivariate logistic regression analysis showed that working mothers (AOR = 0.29; 95% CI 0.12, 0.72), those with no past exclusive breastfeeding experience (AOR = 0.23; 95% CI 0.09, 0.58), those who were offered readymade liquid formula in hospital (AOR = 0.33; 95% CI 0.15, 0.72) and those who lives with their relatives (AOR = 0.21; 95% CI 0.05, 0.81) are less likely to exclusively breastfeed their children for 6 months. (Table 8). On the other hand, mothers with female children (AOR = 2.42; 95% CI 1.18, 4.97) and better breastfeeding knowledge scores (AOR = 1.25; 95% CI 1.04, 1.50) were more likely to exclusively breastfeed their children for 6 months (Table 8).

### Mothers’ breastfeeding knowledge, attitudes, and practices

**Fig. 1:**
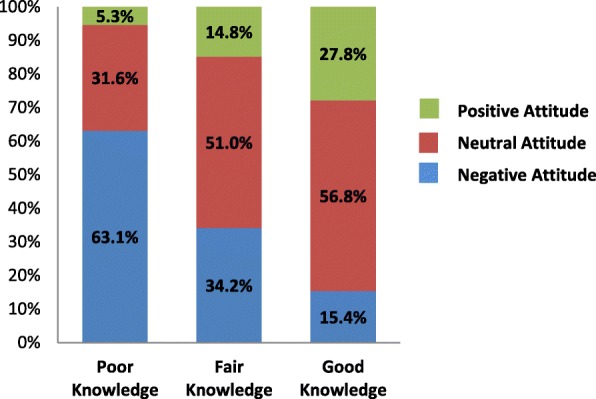
Breastfeeding attitude was affected by breastfeeding knowledge among participants (*n* = 344; *p* = 0.000)

**Fig. 2:**
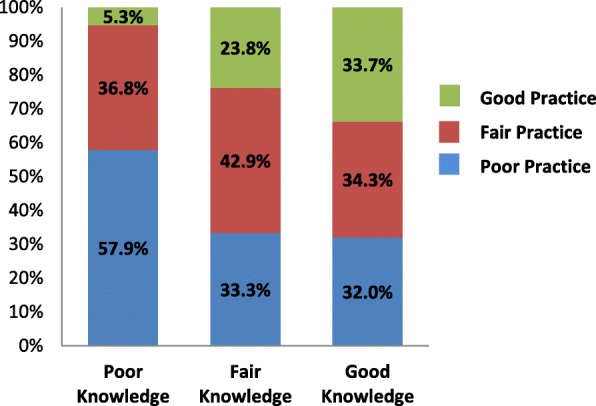
Breastfeeding practice was affected by breastfeeding knowledge among participants (*n* = 344; *p* = 0.022)

Mothers’ breastfeeding attitude was statistically significantly affected by their breastfeeding knowledge (*p* < 0.001) (Fig. 1). In addition, mothers’ breastfeeding practice was also statistically significantly affected by their breastfeeding knowledge (*p* = 0.022) (Fig. 2). There was no statistically significant relationship between mothers’ breastfeeding attitudes and practices.

### Breastfeeding clause in the U.A.E.’s child rights law (2014)

The participants’ opinions about the proposed breastfeeding clause in the U.A.E.’s Child Rights Law were explored. Although only 32.3% of the participants were aware of the proposed breastfeeding clause, the majority of them agreed/strongly agreed that this clause would have a positive effect on babies (84.3%) and disagreed/strongly disagreed that it would have a negative effect on mothers (59.6%).

## Discussion

In our study, 72.6% of the mothers complied with the WHO recommendations of starting breastfeeding within 1 hour of delivery [4, 17]; this figure was higher than that reported in a Saudi Arabian study (31%) [8]. This difference in breastfeeding practice could reflect efforts by hospitals in Abu Dhabi to achieve baby-friendly practices and establish the ten-step initiative for successful breastfeeding as per WHO [18, 19].

Sustained exclusive breastfeeding for up to 6 months without any supplement was reported by 16.9% of the mothers in our study. This figure was close to that seen in another study in Saudi Arabia (15.9%) [20] and was better than the figures reported in numerous other studies in the region [8, 21] as well as in the U.S.A [22, 23]. However, it was lower than that reported in studies in Uganda (49.8%) [24, 25]. The higher exclusive breastfeeding rate at 6 months in this study compared to Saudi Arabian studies could be explained by the fact that a higher percentage of women in our study (81.2%) believed that breast milk alone was sufficient for the child in the first 6 months of life compared with a lower percentage (28%) in a study in Saudi Arabia [8]. This fact was supported in our study, where it was found that mothers who had better breastfeeding knowledge scores were more likely to practice exclusive breastfeeding for 6 months. The higher exclusive breastfeeding rate in the U.A.E. and Saudi Arabia, compared to the U.S.A., could be further explained by the fact that maternity leave is longer in the U.A.E. and Saudi Arabia than in the U.S.A. and that maternity leave is granted with full salary, unlike in the U.S.A [26]. The latter differences could be due to strong family support in the U.A.E., especially from mothers and mothers-in-law; this support was found to have a major effect on breastfeeding knowledge in our study. In addition, women who lived with their relatives had better breastfeeding knowledge and attitudes, and this could further explain the differences observed. On the other hand, the higher exclusive breastfeeding rates in Uganda could be due to their infant feeding tradition and culture: in some parts of Uganda, breastfeeding is considered the only acceptable way to feed an infant [27], and in other parts, women cannot afford formula milk and instead adopt breastfeeding [25]. Another factor that was found to be important in predicting exclusive breastfeeding in our study was whether mothers were offered readymade liquid formula in the hospital. The mothers who were offered readymade formula liquid while in hospital were less likely to exclusively breastfeed for 6 months. The latter could explain the difference in exclusive breastfeeding rates at 6 months between our study and Saudi Arabia [8], where 41.7% of the mothers in our study (compared with 66.7% in Saudi Arabia) were offered ready-made liquid formula in the hospital. This might reflect the Baby-Friendly approach practiced in U.A.E. hospitals. Interestingly, it was also found in our study that female infants were more likely to be exclusively breastfed for 6 months compared to male infants, which could be explained by the cultural infant feeding practices seen in some areas in the Middle East [28].

According to the participants in our study, the most important reason for initiating breastfeeding was child health (89.8%), followed by religious background (37%). This finding was similar to the comparable findings in a study in Saudi Arabia, wherein the main reasons were identical: child health (43.7%), followed by religious background (17.2%) [20]. These similarities might be explained by the fact that these two countries have similar cultures, beliefs, and religion. Specifically, the connection between breastfeeding and religious tradition is most likely related to the Islamic teachings in the Holy Quran, which state, “And mothers shall breastfeed their children for two whole years, for those who desire to complete the appropriate duration of breastfeeding” [29].

Among women who stopped breastfeeding, breastfeeding was stopped at a mean age of 7.1 ± 5.4 months in our study, which was slightly earlier than that in a study in Saudi Arabia (8.7 ± 7.8) [8]. The most common reason given for stopping breastfeeding in our study was decreased breast milk production (76.4%), which corroborates findings in other studies [8, 30, 31]. It is possible, however, that in many cases, decreased breast milk production was perceived rather than real, as some other studies have found. [32] These other studies’ results could mean that the women mistake normal infant behaviour of frequent feeding and waking at night as evidence of hunger, which is a socially acceptable reason for stopping breastfeeding. The second most common reason found in our study for stopping breastfeeding was the baby seeming hungry or unsatisfied after feedings (33.3%). Work related problems were the second most common reason in a study in Saudi Arabia (38.5%) [8]. Collectively, however, 67.4% of the participants in our study reported that work related problems were involved in the decision to stop breastfeeding and that among these, insufficient maternity leave was the most common (14.5%). In our study, it was found that mothers who were employed were less likely to practice exclusive breastfeeding for 6 months, which is consistent with the findings of several studies [33, 34]. Other studies have shown that inadequate comprehensive maternity leave policies, lack of child care facilities at or near the workplace, rigid time schedules that do not allow for nursing breaks, lack of facilities providing privacy for breast-pumping, and absence of facilities for the refrigeration of pumped breast milk are among the factors that affect breastfeeding practice among working mothers [35, 36]. Maternity leave for working mothers in the U.A.E. is very well outlined; mothers are entitled to 60 days’ leave with full salary, which is also the case in Saudi Arabia. However, in Saudi Arabia, maternity leave can also be extended up to 3 years at 25% of the employee’s salary [37]. The effect of these factors is likely to be the reason why most of our participants had shifted to formula feeding by 6 months (89.4%), a proportion similar to that in a study in Saudi Arabia (91.7%) [8].

Most participants intended to breastfeed future children (92.8%), as in two Saudi Arabian studies (90.1%) [8, 38]. These similarities could be explained by the similarity between the cultures and beliefs of these two countries. Another study has shown that such an attitude is one of the strongest predictors of breastfeeding initiation and duration [31].

Most participants (86%) in our study were aware of the benefits of feeding colostrum to the child, as in two Saudi Arabian studies (89.3%) [8, 39]. In contrast, 77% of the surveyed mothers from the district of Rajasthan, India, discarded their colostrum [40]; this finding is similar to those recently reported in other parts of India where 60% of the studied women still discard colostrum [41]. These differences could be explained by the differences between the cultures and beliefs of the U.A.E. and these Indian regions.

Factors that may limit the generalization of our findings include cultural differences between women residing in Abu Dhabi and those residing outside Abu Dhabi; where there is a possible referral bias to our primary health care clinics as 80% of the sample had higher educational level. Furthermore, possible recall bias in responding to the questionnaire for older children could be another factor. Another limitation is that the questionnaire was long, which led to the failure to report complete data regarding some questions.

## Conclusions

Although breastfeeding knowledge in the U.A.E. was generally good, breastfeeding practice was still suboptimal. Modifiable factors that was found to predict exclusive breastfeeding includes breastfeeding knowledge and mothers’ employment status. The most common reasons for stopping breastfeeding were insufficient breast milk production and the baby seeming unsatisfied or hungry after feeding. Therefore, healthcare providers should provide breastfeeding education to all women during their antenatal follow-up visits, especially women with low educational qualifications and no past breastfeeding experience. Solutions should be provided to overcome the barriers to breastfeeding, especially for working mothers, by providing them with longer maternity leave and paid breaks to continue breastfeeding their children. Fortunately, a new law was recently established and passed in the U.A.E. that extends fully paid maternity leave from 2 months to 3 months, which is a step in the right direction.

## Additional files

Additional file 1: Factors that affect breastfeeding knowledge among participants (*n* = 344). (DOCX 34 kb)

Additional file 2: Factors that affect breastfeeding attitudes among participants (*n* = 344). (DOCX 34 kb)

Additional file 3: Factors that affect breastfeeding practices among participants (*n* = 344). (DOCX 33 kb)

## Abbreviations

AAP: American academy of pediatrics
AOR: Adjusted odds ratio
CI: Confidence interval
EBF: Exclusive breastfeeding
FNC: Federal national council
IQR: Interquartile range
SD: Standard deviation
SKMC: Sheikh Khalifa medical City
SPSS: Statistical package for social sciences
U.A.E.: United Arab Emirates
U.S.A.: United States of America
WHO: World health organization
