# Supplementary material for: Knowledge, attitudes, and practices of breastfeeding among women visiting primary healthcare clinics on the island of Abu Dhabi, United Arab Emirates
Source: Int Breastfeed J. 2018 Jul 3;13:26. doi: 10.1186/s13006-018-0165-x (PMC6029179; doi:10.1186/s13006-018-0165-x)
Supplement: Supplementary file 1 — Factors that affect breastfeeding knowledge among participants (n = 344). (DOCX 34 kb) [file 13006_2018_165_MOESM1_ESM.docx]

**Additional file 1: Factors that affect breastfeeding knowledge among participants (n = 344)**

| Variable | Poor  knowledge | Fair knowledge | Good knowledge | p-value |
| --- | --- | --- | --- | --- |
|  | **Number (%)** | **Number (%)** | **Number (%)** |  |
| Age (in years) |  |  |  | 0.126 |
| 18–24 | 4 (10.3) | 21 (53.8) | 14 (35.9) |  |
| 25–29 | 7 (5.5) | 57 (44.5) | 64 (50) |  |
| 30–34 | 2 (1.9) | 39 (36.4) | 66 (61.7) |  |
| 35–39 | 5 (8.8) | 25 (43.8) | 27 (47.4) |  |
| 40–44 | 0 (0) | 5 (50) | 5 (50) |  |
| ≥45 | 0 (0) | 0 (0) | 0 (0) |  |
| Educational background |  |  |  | 0.001 |
| Primary school or lower | 1 (33.3) | 1 (33.3) | 1 (33.4) |  |
| Secondary school | 8 (12.3) | 34 (52.3) | 23 (35.4) |  |
| University or higher | 8 (3) | 113 (41.5) | 151 (55.5) |  |
| Employed |  |  |  | 0.001 |
| No | 10 (4.2) | 118 (49.8) | 109 (46) |  |
| Yes | 8 (7.6) | 30 (28.6) | 67 (63.8) |  |
| Self-employed | 0 (0) | 0 (0) | 0 (0) |  |
| Employment sector |  |  |  | 0.472 |
| Private | 3 (4.4) | 22 (32.4) | 43 (63.2) |  |
| Public | 3 (8.5) | 8 (23) | 24 (68.5) |  |
| Entitled to breastfeeding hours by employer |  |  |  | 0.573 |
| Yes | 6 (7.2) | 22 (26.5) | 55 (66.3) |  |
| No | 1 (5.6) | 7 (38.8) | 10 (55.6) |  |
| Living with husband and children only |  |  |  | 0.000 |
| Yes | 13 (4.4) | 131 (44.7) | 149 (50.9) |  |
| No (Living with  relatives) | 2 (4.7) | 16 (37.2) | 25 (58.1) |  |
| No (Separated/  Divorced/Widowed) | 2 (66.7) | 0 (0) | 1 (33.3) |  |
| Number of housemaids or nannies |  |  |  | 0.170 |
| 0 | 13 (5.4) | 104 (43.3) | 123 (51.3) |  |
| 1 | 1 (1.4) | 25 (35.7) | 44 (62.9) |  |
| >1 | 2 (11.1) | 9 (50) | 7 (38.9) |  |
| Monthly family income (in AED) |  |  |  | 0.011 |
| <15,000 | 10 (7.3) | 71 (51.8) | 56 (40.9) |  |
| 15,000–30,000 | 5 (3.3) | 59 (38.5) | 89 (58.2) |  |
| >30,000 | 1 (2.8) | 11 (30.5) | 24 (66.7) |  |
| Number of children |  |  |  | 0.016 |
| 1 | 5 (4.1) | 50 (41) | 67 (54.9) |  |
| 2–4 | 9 (4.4) | 91 (44.2) | 106 (51.4) |  |
| ≥5 | 3 (25) | 6 (50) | 3 (25) |  |

| Variable | Poor  knowledge | Fair knowledge | Good knowledge | p-value |
| --- | --- | --- | --- | --- |
|  | **Number (%)** | **Number (%)** | **Number (%)** |  |
| Gender of last child |  |  |  | 0.689 |
| Male | 11 (6.5) | 71 (42) | 87 (51.5) |  |
| Female | 7 (4.4) | 70 (43.8) | 83 (51.8) |  |
| Last child’s gestational age at delivery |  |  |  | 0.003 |
| <37 weeks | 8 (11.6) | 32 (46.4) | 29 (42) |  |
| ≥37 weeks | 7 (2.6) | 114 (42.7) | 146 (54.7) |  |
| Mode of delivery of last child |  |  |  | 0.569 |
| Vaginal delivery | 8 (4.1) | 88 (44.9) | 100 (51) |  |
| Caesarian section | 9 (6.2) | 59 (41) | 76 (52.8) |  |
| Healthcare provider explained the importance of breastfeeding during antenatal visits for last pregnancy |  |  |  | 0.049 |
| Yes | 11 (4) | 113 (41.1) | 151 (54.9) |  |
| No | 5 (8.1) | 33 (53.2) | 24 (38.7) |  |
| Healthcare provider explained the importance of breastfeeding after delivery of last child |  |  |  | 0.001 |
| Yes | 11 (3.8) | 114 (39.9) | 161 (56.3) |  |
| No | 4 (7.5) | 34 (64.2) | 15 (28.3) |  |
| Healthcare provider explained the appropriate practices of breastfeeding for last child |  |  |  | 0.000 |
| Yes | 9 (3.4) | 104 (38.8) | 155 (57.8) |  |
| No | 6 (8.8) | 41 (60.3) | 21 (30.9) |  |
| Past breastfeeding experience |  |  |  | 0.002 |
| No other children | 5 (4.3) | 47 (40.2) | 65 (55.5) |  |
| No | 4 (17.4) | 15 (65.2) | 4 (17.4) |  |
| Yes | 6 (3.2) | 83 (44.9) | 96 (51.9) |  |
| Past exclusive breastfeeding experience |  |  |  | 0.169 |
| No other children | 3 (2.8) | 43 (40.2) | 61 (57) |  |
| No | 7 (8.3) | 33 (39.3) | 44 (52.4) |  |
| Yes | 5 (4) | 62 (50) | 57 (46) |  |
