# Supplementary material for: Knowledge, attitudes, and practices of breastfeeding among women visiting primary healthcare clinics on the island of Abu Dhabi, United Arab Emirates
Source: Int Breastfeed J. 2018 Jul 3;13:26. doi: 10.1186/s13006-018-0165-x (PMC6029179; doi:10.1186/s13006-018-0165-x)
Supplement: Supplementary file 2 — Factors that affect breastfeeding attitudes among participants (n = 344). (DOCX 34 kb) [file 13006_2018_165_MOESM2_ESM.docx]

**Additional file 2: Factors that affect breastfeeding attitudes among participants (n=344)**

| Variable | Poor Attitude | Fair Attitude | Good Attitude | p-value |
| --- | --- | --- | --- | --- |
|  | **Number (%)** | **Number (%)** | **Number (%)** |  |
| Age (in years) |  |  |  | 0.003 |
| 18–24 | 14 (35.9) | 21 (53.8) | 4 (10.3) |  |
| 25–29 | 45 (35.2) | 57 (44.5) | 26 (20.3) |  |
| 30–34 | 16 (15) | 70 (65.4) | 21 (19.6) |  |
| 35–39 | 12 (21.1) | 26 (45.6) | 19 (33.3) |  |
| 40–44 | 2 (20) | 6 (60) | 2 (20) |  |
| ≥45 | 0 (0) | 0 (0) | 0 (0) |  |
| Educational background |  |  |  | 0.036 |
| Primary school or lower | 2 (66.7) | 1 (33.3) | 0 (0) |  |
| Secondary school | 25 (38.5) | 29 (44.6) | 11 (16.9) |  |
| University or higher | 60 (22.1) | 151 (55.5) | 61 (22.4) |  |
| Employed |  |  |  | 0.859 |
| No | 62 (26.2) | 127 (53.5) | 48 (20.3) |  |
| Yes | 27 (25.7) | 54 (51.4) | 24 (22.9) |  |
| Self-employed | 0 (0) | 0 (0) | 0 (0) |  |
| Employment sector |  |  |  | 0.017 |
| Private | 21 (30.9) | 29 (42.6) | 18 (26.5) |  |
| Public | 4 (11.4) | 25 (71.4) | 6 (17.2) |  |
| Entitled to breastfeeding hours by employer |  |  |  | 0.703 |
| Yes | 20 (24.1) | 44 (53.0) | 19 (22.9) |  |
| No | 6 (33.3) | 8 (44.5) | 4 (22.2) |  |
| Living with husband and children only |  |  |  | 0.014 |
| Yes | 77 (26.3) | 161 (54.9) | 55 (18.8) |  |
| No (Living with  relatives) | 9 (21) | 17 (39.5) | 17 (39.5) |  |
| No (Separated/  Divorced/Widowed) | 2 (66.7) | 1 (33.3) | 0 (0) |  |
| Number of housemaids or nannies |  |  |  | 0.016 |
| 0 | 64 (26.6) | 124 (51.7) | 52 (21.7) |  |
| 1 | 11 (15.8) | 40 (57.1) | 19 (27.1) |  |
| > 1 | 9 (50) | 9 (50) | 0 (0) |  |
| Monthly family income (in AED) |  |  |  | 0.005 |
| <15,000 | 49 (35.7) | 62 (45.3) | 26 (19) |  |
| 15,000–30,000 | 27 (17.7) | 86 (56.2) | 40 (26.1) |  |
| >30,000 | 8 (22.2) | 23 (63.9) | 5 (13.9) |  |
| Number of children |  |  |  | 0.129 |
| 1 | 32 (26.2) | 65 (53.3) | 25 (20.5) |  |
| 2–4 | 49 (23.8) | 114 (55.3) | 43 (20.9) |  |
| ≥5 | 6 (50) | 2 (16.7) | 4 (33.3) |  |

| Variable | Poor Attitude | Fair Attitude | Good Attitude | p-value |
| --- | --- | --- | --- | --- |
|  | **Number (%)** | **Number (%)** | **Number (%)** |  |
| Gender of last child |  |  |  | 0.368 |
| Male | 39 (23.1) | 96 (56.8) | 34 (20.1) |  |
| Female | 46 (28.8) | 79 (49.4) | 35 (21.8) |  |
| Last child’s gestational age at delivery |  |  |  | 0.005 |
| <37 weeks | 28 (40.6) | 29 (42) | 12 (17.4) |  |
| ≥37 weeks | 57 (21.3) | 150 (56.2) | 60 (22.5) |  |
| Mode of delivery of last child |  |  |  | 0.787 |
| Vaginal delivery | 50 (25.5) | 102 (52.1) | 44 (22.4) |  |
| Caesarian section | 37 (25.7) | 79 (54.9) | 28 (19.4) |  |
| Healthcare provider explained the importance of breastfeeding during antenatal visits for last pregnancy |  |  |  | 0.192 |
| Yes | 72 (26.2) | 140 (50.9) | 63 (22.9) |  |
| No | 14 (22.6) | 39 (62.9) | 9 (14.5) |  |
| Healthcare provider explained the importance of breastfeeding after delivery of last child |  |  |  | 0.423 |
| Yes | 69 (24.1) | 154 (53.9) | 63 (22) |  |
| No | 17 (32.1) | 27 (50.9) | 9 (17) |  |
| Healthcare provider explained the appropriate practices of breastfeeding for last child |  |  |  | 0.634 |
| Yes | 64 (23.9) | 147 (54.8) | 57 (21.3) |  |
| No | 20 (29.4) | 34 (50) | 14 (20.6) |  |
| Past breastfeeding experience |  |  |  | 0.858 |
| No other children | 31 (26.5) | 62 (53) | 24 (20.5) |  |
| No | 7 (30.4) | 12 (52.2) | 4 (17.4) |  |
| Yes | 41 (22.2) | 103 (55.6) | 41 (22.2) |  |
| Past exclusive breastfeeding experience |  |  |  | 0.620 |
| No other children | 27 (25.3) | 56 (52.3) | 24 (22.4) |  |
| No | 18 (21.4) | 52 (61.9) | 14 (16.7) |  |
| Yes | 31 (25) | 64 (51.6) | 29 (23.4) |  |
