# Supplementary material for: Knowledge, attitudes, and practices of breastfeeding among women visiting primary healthcare clinics on the island of Abu Dhabi, United Arab Emirates
Source: Int Breastfeed J. 2018 Jul 3;13:26. doi: 10.1186/s13006-018-0165-x (PMC6029179; doi:10.1186/s13006-018-0165-x)
Supplement: Supplementary file 3 — Factors that affect breastfeeding practices among participants (n = 344). (DOCX 33 kb) [file 13006_2018_165_MOESM3_ESM.docx]

| Variable | Poor Practice | Fair Practice | Good Practice | p-value |
| --- | --- | --- | --- | --- |
|  | **Number (%)** | **Number (%)** | **Number (%)** |  |
| Age (in years) |  |  |  | 0.617 |
| 18–24 | 17 (44.7) | 11 (29) | 10 (26.3) |  |
| 25–29 | 41 (32.5) | 49 (38.9) | 36 (28.6) |  |
| 30–34 | 31 (29.8) | 41 (39.4) | 32 (30.8) |  |
| 35–39 | 23 (40.3) | 20 (35.1) | 14 (24.6) |  |
| 40–44 | 2 (20) | 6 (60) | 2 (20) |  |
| ≥45 | 0 (0) | 0 (0) | 0 (0) |  |
| Education background |  |  |  | 0.359 |
| Primary school or lower | 2 (66.7) | 1 (33.3) | 0 (0) |  |
| Secondary school | 27 (42.2) | 22 (34.4) | 15 (23.4) |  |
| University or higher | 85 (31.8) | 103 (38.6) | 79 (29.6) |  |
| Employed |  |  |  | 0.443 |
| No | 75 (32.3) | 88 (38) | 69 (29.7) |  |
| Yes | 40 (38.5) | 39 (37.5) | 25 (24.0) |  |
| Self-employed | 0 (0) | 0 (0) | 0 (0) |  |
| Employment sector |  |  |  | 0.743 |
| Private | 25 (37.3) | 24 (35.8) | 18 (26.9) |  |
| Public | 14 (40) | 14 (40) | 7 (20) |  |
| Entitled to breastfeeding hours by employer |  |  |  | 0.954 |
| Yes | 31 (37.8) | 30 (36.6) | 21 (25.6) |  |
| No | 7 (38.9) | 7 (38.9) | 4 (22.2) |  |
| Living with husband and children only |  |  |  | 0.699 |
| Yes | 97 (33.8) | 107 (37.3) | 83 (28.9) |  |
| No (Living with  relatives) | 14 (32.5) | 18 (41.9) | 11 (25.6) |  |
| No (Separated/  Divorced/Widowed) | 2 (66.7) | 1 (33.3) | 0 (0) |  |
| Number of housemaids or nannies |  |  |  | 0.996 |
| 0 | 81 (34.5) | 89 (37.9) | 65 (27.6) |  |
| 1 | 24 (34.3) | 25 (35.7) | 21 (30) |  |
| >1 | 6 (33.3) | 7 (38.9) | 5 (27.8) |  |
| Monthly family income (in AED) |  |  |  | 0.112 |
| <15,000 | 54 (40.3) | 50 (37.3) | 30 (22.4) |  |
| 15,000–30,000 | 40 (26.7) | 59 (39.3) | 51 (34) |  |
| >30,000 | 13 (36.1) | 13 (36.1) | 10 (27.8) |  |
| Number of children |  |  |  | 0.151 |
| 1 | 51 (42.5) | 40 (33.3) | 29 (24.2) |  |
| 2–4 | 59 (29.2) | 81 (40.1) | 62 (30.7) |  |
| ≥5 | 5 (41.7) | 5 (41.7) | 2 (16.6) |  |

| Variable | Poor Practice | Fair Practice | Good Practice | p-value |
| --- | --- | --- | --- | --- |
|  | **Number (%)** | **Number (%)** | **Number (%)** |  |
| Gender of last child |  |  |  | 0.196 |
| Male | 60 (36.1) | 67 (40.4) | 39 (23.5) |  |
| Female | 51 (32.5) | 55 (35) | 51 (32.5) |  |
| Last child gestational age at delivery |  |  |  | 0.162 |
| <37 weeks | 27 (39.7) | 28 (41.2) | 13 (19.1) |  |
| ≥37 weeks | 84 (32.1) | 98 (37.4) | 80 (30.5) |  |
| Mode of delivery of last child |  |  |  | 0.003 |
| Vaginal delivery | 83 (27.6) | 74 (38.5) | 65 (33.9) |  |
| Caesarian section | 61 (43) | 53 (37.3) | 28 (19.7) |  |
| Healthcare provider explained the importance of breastfeeding during antenatal visits for last pregnancy |  |  |  | 0.445 |
| Yes | 93 (34.3) | 99 (36.5) | 79 (29.2) |  |
| No | 19 (31.7) | 27 (45) | 14 (23.3) |  |
| Healthcare provider explained the importance of breastfeeding after delivery of last child |  |  |  | 0.821 |
| Yes | 93 (33) | 110 (39) | 79 (28) |  |
| No | 19 (37.3) | 18 (35.3) | 14 (27.4) |  |
| Healthcare provider explained the appropriate practices of breastfeeding for last child |  |  |  | 0.497 |
| Yes | 85 (32.2) | 101 (38.3) | 78 (29.5) |  |
| No | 25 (37.9) | 26 (39.4) | 15 (22.7) |  |
| Past breastfeeding experience |  |  |  | 0.064 |
| No other children | 46 (39.7) | 41 (35.3) | 29 (25) |  |
| No | 10 (45.5) | 9 (40.9) | 3 (13.6) |  |
| Yes | 48 (26.4) | 75 (41.2) | 59 (32.4) |  |
| Past exclusive breastfeeding experience |  |  |  | 0.009 |
| No other children | 43 (40.6) | 37 (34.9) | 26 (24.5) |  |
| No | 27 (32.9) | 40 (48.8) | 15 (18.3) |  |
| Yes | 32 (26.2) | 44 (36.1) | 46 (37.7) |  |
|  |  |  |  |  |

**Additional file 3: Factors that affect breastfeeding practices among participants (n=344)**
